# Resveratrol ameliorates maternal separation-induced anxiety- and depression-like behaviors and reduces Sirt1-NF-kB signaling-mediated neuroinflammation

**DOI:** 10.3389/fnbeh.2023.1172091

**Published:** 2023-05-18

**Authors:** Ru-Meng Wei, Yue-Ming Zhang, Yi-Zhou Feng, Kai-Xuan Zhang, Jing-Ya Zhang, Jing Chen, Bao-Ling Luo, Xue-Yan Li, Gui-Hai Chen

**Affiliations:** Department of Neurology (Sleep Disorders), The Affiliated Chaohu Hospital of Anhui Medical University, Hefei, Anhui, China

**Keywords:** resveratrol, Sirt1, maternal separation, anxiety, depression, NF-κB

## Abstract

Maternal separation in early life has a detrimental effect on the physiological and biochemical functions of the brains of offspring and can lead to anxiety- and depression-like behaviors later in life. Resveratrol possesses a variety of pharmacological properties, including anti-inflammatory, anxiolytic, and anti-depressive effects. In rodents, resveratrol can attenuate anxiety- and depression-like behaviors induced by chronic unpredictable mild stress, estrogen deficiency, and lipopolysaccharide. However, whether resveratrol administration during adolescence can counteract these behaviors when they result from maternal separation is unknown. In this study, male C57BL/6J mice were separated from their mothers for 4 h per day from postnatal day 2 (PND 2) to PND 21; starting on PND 61, resveratrol was administered intraperitoneally at 40 mg/(kg/day^–1^) for 4 weeks. At 3 months of age, anxiety and depression-like behaviors were assessed in the male offspring using a series of tasks consisting of an open field test, an elevated plus maze test, a forced swimming test, and a tail suspension test. The hippocampal levels of interleukin (IL)-1β, IL-6, and tumor necrosis factor-alpha (TNF-α) were measured by ELISA, while those of sirtuin 1 (Sirt1) and nuclear factor kappa B (NF-κB) p65 were determined by western blotting and PCR. The results showed that maternal separation led to increased anxiety- and depression-like behaviors, enhanced the levels of pro-inflammatory cytokines, and downregulated the Sirt1/NF-κB signaling pathway in the male offspring; however, these effects could be reversed by treatment with resveratrol. Our findings suggested that resveratrol can ameliorate inflammation and anxiety- and depression-like behaviors induced by maternal separation via the activation of the Sirt1/NF-κB pathway.

## 1. Introduction

Early life experiences have a profound influence on the development of mental health later in life (McEwen, 2008). Stress exposure early in life has a detrimental effect on brain development, increasing the risk of psychiatric disorders, aggression, and substance abuse in adulthood (Andersen, 2015). Early human life stressors include child abuse, exposure to violence, traumatic events, and neglect (Mandelli et al., 2015; Infurna et al., 2016; Li M. et al., 2016; LeMoult et al., 2020). Maternal separation in early life of rodent animals are thought to mimic well the disruption in normal mother-child interactions in humans (Fang et al., 2023). Numerous studies have used animal models of mother-child separation to investigate the pathophysiological mechanisms of emotional disorders induced by early life stress (Jiang et al., 2021; Qin et al., 2021; Wang et al., 2022). Current evidence indicates that maternal separation alters neuroendocrine signaling, synaptic plasticity, and neuronal morphology in offspring. This can lead to psychiatric disorders during adolescence and adulthood, including anxiety, depression, and cognitive impairment, which severely affect long-term quality of life and impose a significant economic burden on society (Fabricius et al., 2008; Tractenberg et al., 2016; Cui et al., 2020). Accordingly, therapeutic options for the treatment or prevention of stress-related anxiety and depression early in life are urgently required.

It is increasingly clear that inflammation plays an important role in the pathophysiology of anxiety and depression (Zheng et al., 2021). Early life stress induces inflammatory processes in the central nervous system, accompanied by the release of pro-inflammatory cytokines such as interleukin-1 beta (IL-1β), IL-6, and tumor necrosis factor-alpha (TNF-α) (Andersen, 2022). These factors can induce inflammatory responses in the brain through specific immune-brain signaling pathways, resulting in many behavioral, neurochemical, and hormonal changes following exposure to stress (Ganguly and Brenhouse, 2015). In humans, the levels of pro-inflammatory cytokines IL-1β, IL-6, and TNF-α were reported to be significantly higher in the post-mortem brains of suicided patients with depression than in those of matched non-psychiatric controls (Pandey, 2017). In mice, meanwhile, early life stress was shown to increase the concentrations of pro-inflammatory cytokines both in the periphery and the central nervous system as well as induce anxiety and depression-like behaviors (Mi et al., 2022).

The hippocampus is an important region not only associated with learning and memory, but also the mesolimbic system involved in emotion regulation, and its function is vulnerable to impairment by adverse stress (Bannerman et al., 2004; Grigoryan and Segal, 2016; Tang et al., 2019). Studies have shown that hippocampal neuroinflammation response is an important pathogenetic mechanism involved in stress-induced anxiety and depression disorders early in life (Bachiller et al., 2020). For example, substantial evidence suggests that maternal separation can lead to increased anxiety- and depression-like behavior and hippocampal inflammatory response in rodents (Wang et al., 2017; Zhou et al., 2020; Mi et al., 2022). These observations suggest that inflammation response may be the underlying mechanism through which maternal separation induces anxiety and depression in offspring. Accordingly, it is essential to identify remedy that can reverse inflammation response for the treatment of stress-induced anxiety and depression-like behaviors early in life.

Resveratrol (3,4′,5-trihydroxy-*trans-*stilbene) is a polyphenol with antioxidant properties that is naturally found in the skins of red grapes, mulberries, Japanese knotweed, and some nuts (Gambini et al., 2015). In addition, it has pharmacological properties including anti-inflammatory, anti-aging, anti-cancer (Sakr et al., 2015) and neuroprotective effects. Indeed, Surya et al. (2022) reported that resveratrol can promote neurogenesis, thereby slowing the progression of Alzheimer’s disease. Evidence supports that resveratrol exerts anxiolytic and anti-depressive effects in part through the activation of sirtuin 1 (Sirt1), a mediator of inflammation initiation and progression. Sirt1 activation reduces the levels of pro-inflammatory cytokines, eventually leading to the amelioration of stress-induced neuroinflammation and depression-like behaviors in rodents (Ge et al., 2016; Kim et al., 2016; Lu et al., 2018; Yu et al., 2019). For example, resveratrol can counteract anxiety- and depression-like behaviors induced by estrogen deficiency in adult female C57BL/6J mice by inhibiting pro-inflammatory processes in the hippocampus via the activation of the Sirt1/NF-κB signaling pathway (Liu et al., 2019). Similarly, polydatin, a derivative of resveratrol, can improve lipopolysaccharide-induced depression-like behavior in adult male mice primarily through the Sirt1/NF-κB signaling pathway (Bian et al., 2022). Combined, these findings suggest that the pharmacological activation of Sirt1 may be a promising therapeutic strategy for inflammation-induced, depression-associated phenotypes (Salminen et al., 2008; Yao and Rahman, 2012; Xie et al., 2013). However, whether resveratrol can ameliorate maternal separation-induced anxiety- and depression-like behaviors remains largely unexplored.

In the present study, it was considered that hormone level fluctuations during the estrous cycle of female mice may interfere with the results of behavioral experiments (Wei et al., 2018; Zhou et al., 2020; Kim et al., 2022), the male C57BL/6J mice were used to investigate whether resveratrol can ameliorate maternal separation-induced anxiety- and depression-like behaviors in adulthood, and, if so, whether the mechanism involves the inhibition of inflammation response through the modulation of the Sirt1/NF-κB signaling pathway.

## 2. Materials and methods

### 2.1. Animals

Ten-week-old female and male C57BL/6J mice were purchased from the Beijing Vital River Laboratory Animal Company (Shanghai, China). The mice were housed in a standard environment (temperature: 22 ± 1°C; relative humidity: 55 ± 5%; 12/12 h light/dark cycle, with lights on at 07:00) for 2 weeks with *ad libitum* access to food and water. After 2 weeks of acclimatization, the animals were mated (one male with two females). The delivery day was designated as postnatal day 0 (PND 0). The subjects of the study were male offspring mice. The study was conducted in compliance with the guidelines established by the National Institutes of Health Guide for the Care and Use of Laboratory Animals (National Research Council (US), 2011), the Association of Laboratory Animal Sciences and the Center for Laboratory Animal Sciences at Anhui Medical University (approval number: LLSC20190710) and “ARRIVE guidelines.^1^”

### 2.2. Separation procedure

The newborn pups were randomly divided into a non-maternal separation group (NMS) and a maternal separation (MS) group. In the MS group, the pups were separated from their mothers for 4 h per day (10:00 to 14:00 h) from PND 2 to PND 21 (Huang et al., 2021). During this period, the pups were placed in clean cages containing bedding material placed on a heating pad. In the NMS group, all the pups stayed with their mothers until weaning.

### 2.3. Experimental groups

**FIGURE 1:**
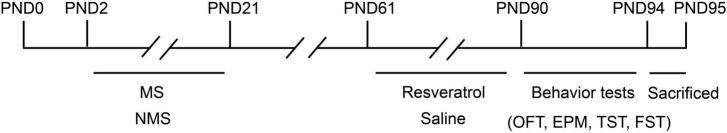
Timeline of the experiment (refer experimental protocols for details).

The animals were randomly assigned to one of the following four groups, 8 mice per group, total 32 mice: A control (CON) group, a control + resveratrol (CR) group, a MS group, and a MS + resveratrol (MSR) group. Mice in the CR and MSR groups received resveratrol [40 mg/(kg/day^–1^)] for 4 weeks by intraperitoneal injection (Shen et al., 2019). Resveratrol (Absin, abs815905, white crystalline powder, purity >98%) was dissolved in a 90% saline solution containing 5% DMSO (Absin, abs9189) and 5% Tween 80 (Abbexa, abx082610). The control and MS groups received the same volume of saline containing 5% DMSO and 5% Tween 80 but no resveratrol (Figure 1). All behavioral tests were conducted in accordance with double-blind methods. No animals were excluded throughout the experiment. The sample size of experimental animals was selected based on the previous studies of our group (Wei et al., 2022).

### 2.4. Open field test

The open field test was used to assess anxiety-like behavior in the mice at PND 91. In this test, decreases in the time spent in the central region and the number of entries into the central region are considered to represent an increase in anxiety-like behavior in mice (Li X. Y. et al., 2016). The open field under dim lights (30 lx) consisted of an open square box 50 cm long × 50 cm wide × 25 cm high. The mice were placed in the center of the box and allowed to freely explore for 5 min. After each mouse, the box was washed with 75% alcohol and then air-dried to eliminate the effect of the odor of the last mouse. The time spent in the central region, the number of entries into the central region, and the total distance traveled during the 5 min were recorded for each group of mice using ANY-Maze software (Stoeling, USA).

### 2.5. Elevated plus maze test

The elevated plus maze test was administered at PND 92 using a procedure similar to that described in Wei et al. (2022). A decrease in the time spent in, and the number of entries into, the open arms are thought to be associated with increased anxiety-like behavior in mice. The apparatus under illumination (30 lx) consisted of a cross-shaped platform containing two opposing open arms (each 30 cm long × 6 cm wide), two opposing closed arms (each 30 cm long × 6 cm × 15 cm high), and a central arena (6 cm × 6 cm). The apparatus was raised 80 cm above the ground. The mice were placed on the central platform facing one of the open arms and allowed to freely explore the maze for 6 min. The apparatus was washed with 75% alcohol after each mouse to eliminate odor interference. ANY-Maze software was used to record the time spent in, and the number of entries into, the open arms.

### 2.6. Tail suspension test

The tail suspension test was performed as previously described at PND 93 (Ueno et al., 2022). In this test, an increase in immobility time is thought to reflect an increase in depression-like behavior in mice. Tape was wrapped around the tail of each mouse, approximately 1 cm from the tip, after which the mouse was suspended from a metal hook using the tape, with the nose tip of the animal approximately 35 cm above the ground. The tail of the mouse was passed through a plastic cylinder to prevent tail climbing during the test. The behavior of each animal was recorded for 6 min using video, with the final 4 min being used for quantification. Immobility time during the last 4 min was monitored by a blinded observer.

### 2.7. Forced swimming test

To further evaluate the depression-like behavior in each group of mice, a forced swimming test was used at PND 94. The test was performed in a glass cylindrical container (28 cm in height, 18 cm in diameter) filled with clean water (22 ± 1°C) to a depth of 15 cm. The whole 6 min of the experiment was recorded using video. The immobility time in the final 4 min was used for quantification by a blinded observer. The animals were considered to be immobile when they were floating passively or making only those movements necessary for maintaining balance.

### 2.8. Tissue preparation

Mice were euthanized using 2% sodium pentobarbital in autopsy laboratory at PND95. Hippocampal tissue was quickly isolated from the brain and snap frozen in liquid nitrogen, and subsequently stored in a −80°C refrigerator. When needed, samples were taken out for biochemical analyses.

### 2.9. Measurement of cytokine levels

The samples were homogenized, centrifuged at 2,000–3,000 rpm for 20 min, and the supernatants were collected. The levels of IL-1β, IL-6, and TNF-a in the supernatants were quantified using mouse- and cytokine-specific ELISA kits [Wuhan Colorful Gene Biotechnology Co. (JYM0531Mo, JYM0012Mo, JYM0218Mo)] according to the manufacturer’s instructions. The results were expressed as optical density. Eight samples were analyzed per group.

### 2.10. Real-time fluorescence-based quantitative PCR

Total RNA was extracted from hippocampal tissue using TRIzol (Life Technologies, 15596018) as previously described (Zhang et al., 2022). The isolated RNA was reverse transcribed to cDNA using a reverse transcription kit (TaKaRa, RR047A). The cDNA was subjected to qPCR using primers targeting NF-κB p65, Sirt1, and β-actin. The sequences of the primers used are listed in Table 1. The PCR mixture contained 5 μL of 2 × SYBR Green Mixture, 1 μL of forward primer, 1 μL of reverse primer, 1 μL of cDNA, and 2 μL of RNase-free water. The cycling parameters were one cycle of 95°C for 60 s, followed by 40 cycles of 95°C for 20 s and 60°C for 60 s. Relative expression levels were calculated using the 2^–ΔΔ*Ct*^ method, which was based on the formula (ΔΔCt = ΔCt sample—ΔCt reference) as described previously (Livak and Schmittgen, 2001; Wei et al., 2022).

**TABLE 1:** The sequences of the primers used for qPCR.

Gene	Amplicon size (bp)	Forward primer (5′→3′)	Reverse primer (5′→3′)
β-actin	120	AGTGTGACGTTGACATCCGT	TGCTAGGAGCCAGAGCAGTA
NF-κB p65	119	GCTCCTGTTCGAGTCTCCAT	TTGCGCTTCTCTTCAATCCG
Sirt1	116	TAATGTGAGGAGTCAGCACC	GCCTGTTTGGACATTACCAC

### 2.11. Western blotting

Western blotting was performed as described by Zhang et al. (2022). Briefly, total protein was extracted from hippocampal tissue using RIPA lysis buffer (Beyotime, P0013B). The supernatant containing the protein was collected following centrifugation at 12,000 × *g* for 15 min. Then, 5 × SDS–PAGE loading buffer was added to the protein samples in a 1:4 ratio, followed by boiling for 15 min to fully denature the proteins. After cooling to room temperature, the proteins were separated by SDS-PAGE (constant pressure, 80 V electrophoresis for 1 h), and transferred to PVDF membranes (membrane transfer times of 50 min for NF-κB p65, 50 min for acetylated-NF-κB p65, and 70 min for Sirt1). After the transfer is completed, the protein membrane is immediately placed into the pre-prepared Western wash solution and rinsed for 5 min to wash off the transfer membrane solution from the membrane. Add Western Closure Solution (5% skim milk powder), shake slowly on a shaker, and close the membrane for 2 h at room temperature. The membranes were then incubated overnight at 4°C with the following diluted primary antibodies mouse anti-Sirt1 antibody (1:4,000, Abcam, ab110304), mouse anti-NF-κB p65 antibody (1:2,000, Proteintech, 66535-1-Ig), and rabbit anti-acetyl-NF-κB p65 antibody (1:1,000, Abcam, ab237591) according to the manufacturer’s instructions. After three washes with PBST, the samples were incubated with secondary antibodies (HRP-labeled goat anti-rabbit IgG, 1:1:20,000, Zsbio, ZB-2301; goat anti-mouse IgG, 1:20,000, Zsbio, ZB-2305) at room temperature for 1.2 h. Finally, protein bands were detected using an ECL ultrasensitive luminescence kit (Thermo, 340958) and analyzed using ImageJ software (Media Cybernetics, USA). GAPDH was used as internal controls. The gray value of the target protein is divided by the gray value of the internal reference protein for normalization.

### 2.12. Statistical analysis

All data were analyzed using GraphPad Prism version 8.0. Normally distributed data are reported as means ± standard error of the mean (SEM). Differences among the groups were tested using two-way analysis of variance (ANOVA) with treatment and drug as independent variables followed by Tukey’s *post-hoc* multiple comparison test. *P*-values < 0.05 were considered significant.

## 3. Results

### 3.1. Resveratrol ameliorated maternal separation-induced anxiety-like behaviors in male offspring

**FIGURE 2:**
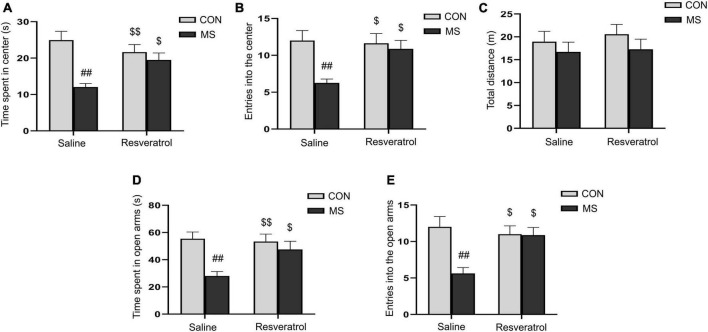
Resveratrol treatment ameliorated maternal separation-induced anxiety-like behaviors in mice. Anxiety-like behaviors were assessed using the open field test **(A–C)** and the elevated plus maze test **(D,E)**. **(A)** Time spent in the central region in the open field test. **(B)** Number of entries into the central region in the open field test. **(C)** Total distance traveled in the open field test. **(D)** Time spent in the open arms in the elevated plus maze test. **(E)** Number of entries into the open arms in the elevated plus maze test. All data are shown as means ± SEM. ^##^*P* < 0.01 vs. the CON group; ^$^*P* < 0.05, ^$$^*P* < 0.01 vs. the MS group. *N* = 8 per group. CON, control; MS, maternal separation.

We assessed the level of anxiety-like behaviors in each group of mice using the open-field and elevated maze tests. In the open field test, two-way ANOVA showed that a significantly effect of treatment [time spent in the central region: *F*_(1, 28)_ = 15.353, *P* < 0.01; number of entries into the central region: *F*_(1, 28)_ = 8.041, *P* < 0.01] and interaction of treatment × drug [time spent in the central region: *F*_(1, 28)_ = 7.891, *P* < 0.01; number of entries into the central region: *F*_(1, 28)_ = 4.758, *P* = 0.038], but no drug [time spent in the central region: *F*_(1, 28)_ = 1.180, *P* = 0.287; number of entries into the central region: *F*_(1, 28)_ = 3.438, *P* = 0.074], on the time spent in the central region and the number of entries into the central region (Figures 2A, B). *Post hoc* analysis demonstrated that maternal separation resulted in a significant reduction in the time spent in the central region, as well as the number of entries into the central region when compared with the control condition (*Ps* < 0.01). However, these effects were alleviated with resveratrol administration (*Ps* < 0.05). No difference in the distance covered in 5 min was observed among the different groups [treatment: *F*_(1, 28)_ = 1.595, *P* = 0.217; drug: *F*_(1, 28)_ = 0.256, *P* = 0.617; treatment × drug: *F*_(1, 28)_ = 0.058, *P* = 0.811] (Figure 2C).

In the elevated plus maze test, there was a significant effect of treatment and interaction of treatment × drug, but no significant effect of drug, in the time spent in the open arms [treatment: *F*_(1, 28)_ = 10.878, *P* < 0.01; drug: *F*_(1, 28)_ = 3.005, *P* = 0.094; treatment × drug: *F*_(1, 28)_ = 4.626, *P* = 0.040] and the number of entries into the open arms [treatment: *F*_(1, 28)_ = 8.309, *P* < 0.01; drug: *F*_(1, 28)_ = 3.552, *P* = 0.070; treatment × drug: *F*_(1, 28)_ = 7.682, *P* < 0.01] (Figures 2D, E). Additionally, mice in the MS group spent less time in and had fewer entries into the open arms compared with control mice (*Ps* < 0.01). As in the open field test, these effects were mitigated with resveratrol administration (*P* < 0.05 for both the time spent in the open arms and the number of entries into the open arms).

### 3.2. Resveratrol ameliorated maternal separation-induced depression-like behavior in male offspring

**FIGURE 3:**
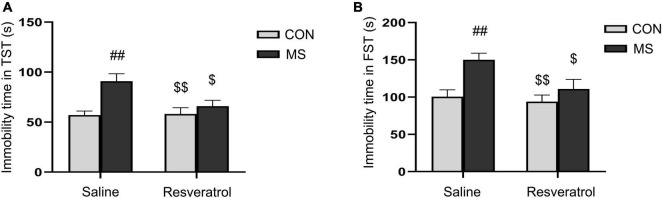
Resveratrol treatment ameliorated maternal separation-induced depression-like behaviors in mice. Depression-like behavior was evaluated using the tail suspension and forced swimming tests. **(A)** Immobility time in the tail suspension test. **(B)** Immobility time in the forced swimming test. All data are shown as means ± SEM. ^##^*P* < 0.01 vs. the CON group; ^$^*P* < 0.05, ^$$^*P* < 0.01 vs. the MS group. *N* = 8 per group. CON, control; MS, maternal separation.

In the tail suspension test, there was a significant effect of treatment and interaction of treatment × drug significant differences, but no drug effect in immobility time [treatment: *F*_(1, 28)_ = 11.780, *P* < 0.01; drug: *F*_(1, 28)_ = 3.875, *P* = 0.060; treatment × drug: *F*_(1, 28)_ = 4.640, *P* = 0.040] (Figure 3A). Immobility time was significantly longer in the MS group than in both the CON group (*P* < 0.01) and the MSR group (*P* < 0.05). However, no differences were seen between mice in the MSR group and control mice (*P* > 0.05). In the forced swimming test, there was a significant effect of treatment [*F*_(1, 28)_ = 10.856, *P* < 0.01] and drug[*F*_(1, 28)_ = 5.194, *P* = 0.031], but no interaction effect [*F*_(1, 28)_ = 2.593, *P* = 0.119] in immobility time (Figure 3B). *Post hoc* analysis showed that immobility time was longer in mice of the MS group than in those of the CON group (*P* < 0.01) or MSR group (*P* < 0.05).

### 3.3. Resveratrol suppressed the maternal separation-induced increase in pro-inflammatory cytokine levels

**FIGURE 4:**
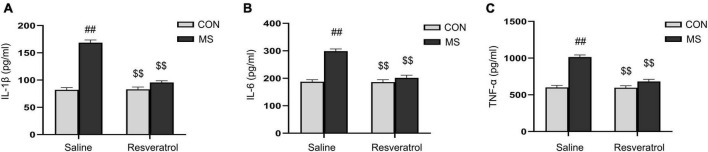
Resveratrol treatment suppressed the maternal separation-induced upregulation of the levels of IL-1β, IL-6, and TNF-α in the mouse hippocampus. **(A)** IL-1β, **(B)** IL-6, and **(C)** TNF-α. All data are shown as means ± SEM. ^##^*P* < 0.01 vs. the CON group; ^$$^*P* < 0.01 vs. the MS group. *N* = 8 per group. CON, control; MS, maternal separation.

A significant effect of treatment, drug, and interaction of treatment × drug were observed in both the hippocampal levels of IL-1β [treatment: *F*_(1, 28)_ = 139.730, *P* < 0.01; drug: *F*_(1, 28)_ = 73.783, *P* < 0.01; treatment × drug: *F*_(1, 28)_ = 77.202, *P* < 0.01], IL-6 [treatment: *F*_(1, 28)_ = 58.605, *P* < 0.01; drug: *F*_(1, 28)_ = 34.979, *P* < 0.01; treatment × drug: *F*_(1, 28)_ = 33.244, *P* < 0.01], and TNF-α [treatment: *F*_(1, 28)_ = 80.802, *P* < 0.01; drug: *F*_(1, 28)_ = 36.335, *P* < 0.01; treatment × drug: *F*_(1, 28)_ = 35.099, *P* < 0.01] (Figures 4A–C). Additionally, the hippocampal levels of the three cytokines were higher in the MS group than in the CON group (*Ps* < 0.01). Nevertheless, resveratrol treatment significantly mitigated this increase (*Ps* < 0.01). No differences in IL-1β, IL-6, and TNF-α contents were observed between the CON and CR groups (*Ps* > 0.05).

### 3.4. The effect of resveratrol on the mRNA levels of Sirt1 and NF-κB p65 in the hippocampus of mice subjected to maternal separation

**FIGURE 5:**
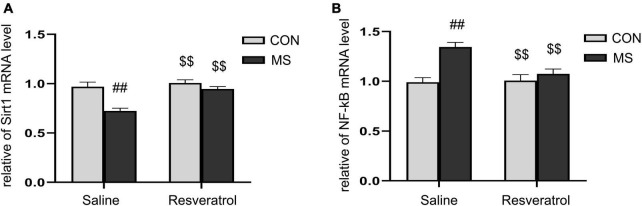
Resveratrol reversed the maternal separation-induced reduction in Sirt1 mRNA expression levels and increase in NF-κB p65 mRNA expression levels in the hippocampus. **(A)** Relative Sirt1 mRNA expression levels. **(B)** Relative NF-κB p65 mRNA expression levels. All data are shown as means ± SEM. ^##^*P* < 0.01 vs. the CON group; ^$$^*P* < 0.01 vs. the MS group. *N* = 8 per group. CON, control; MS, maternal separation.

As shown in Figure 5, there were significant effects of treatment, drug, and interaction of treatment × drug in the mRNA levels of Sirt1 [treatment: *F*_(1, 28)_ = 20.548, *P* < 0.01; drug: *F*_(1, 28)_ = 14.740, *P* < 0.01; treatment × drug: *F*_(1, 28)_ = 7.576, *P* = 0.010] and NF-κB p65 [treatment: *F*_(1, 28)_ = 17.366, *P* < 0.01; drug: *F*_(1, 28)_ = 6.329, *P* = 0.018; treatment × drug: *F*_(1, 28)_ = 8.052, *P* < 0.01] in the hippocampus. *Post hoc* analysis showed that the level of Sirt1 mRNA was significantly lower and that of NF-κB p65 mRNA significantly higher in the MS group than in the CON group (*Ps* < 0.01). Moreover, the mRNA level of Sirt1 was higher and that of NF-κB p65 lower (*Ps* < 0.01) in the MSR group relative to the MS group (Figures 5A, B).

### 3.5. Effect of resveratrol on maternal separation-induced the altered protein levels of Sirt1, NF-κB p65, and acetyl-NF-κB p65 in the hippocampus

**FIGURE 6:**
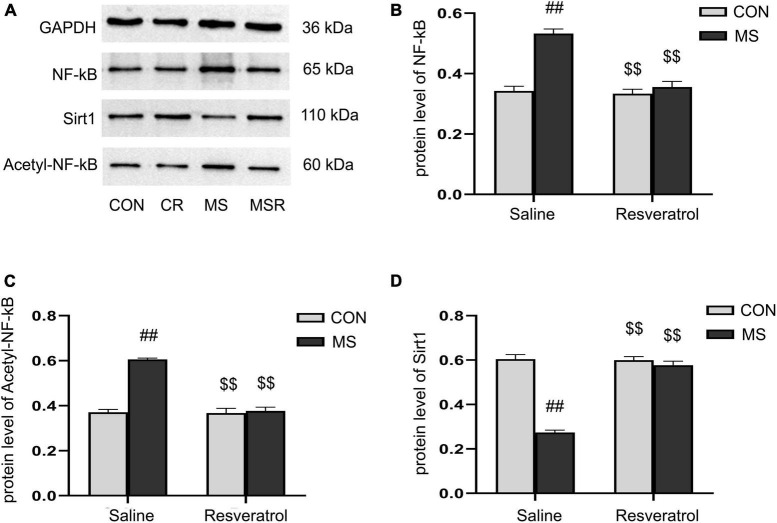
Resveratrol suppressed the maternal separation-induced reduction in the protein levels of Sirt1 and increase in those of NF-κB p65 and acetyl-NF-κB p65 in the hippocampus. **(A)** The protein levels of NF-κB p65, acetyl-NF-κB p65, and Sirt1 in the hippocampus as determined by western blotting. **(B–D)** The results of protein quantification. **(B)** NF-κB p65, **(C)** acetyl-NF-κB p65, and **(D)** Sirt1. All data are shown as means ± SEM. ^##^*P* < 0.01 vs. the CON group; ^$$^*P* < 0.01 vs. the MS group. *N* = 6 per group. CON, control; CR, control + resveratrol; MS, maternal separation; MSR, MS + resveratrol.

A significant effect of treatment, drug, and interaction of treatment × drug was observed in both the protein levels of NF-κB p65 [treatment: *F*_(1, 28)_ = 44.728, *P* < 0.01; drug: *F*_(1, 28)_ = 34.430, *P* < 0.01; treatment × drug: *F*_(1, 28)_ = 27.866, *P* < 0.01], acetyl-NF-κB p65 [treatment: *F*_(1, 28)_ = 65.796, *P* < 0.01; drug: *F*_(1, 28)_ = 59.488, *P* < 0.01; treatment × drug: *F*_(1, 28)_ = 55.954, *P* < 0.01], and Sirt1 [treatment: *F*_(1, 28)_ = 112.067, *P* < 0.01; drug: *F*_(1, 28)_ = 80.168, *P* < 0.01; treatment × drug: *F*_(1, 28)_ = 85.084, *P* < 0.01] in the hippocampus. *Post hoc* analysis showed that the protein levels of NF-κB p65 and acetyl-NF-κB p65 were increased and those of Sirt1 decreased in the MS group compared with those of the CON group (*Ps* < 0.01). Compared to the MS group, the MSR group had significantly decreased NF-κB, acetyl-NF-κB and increased Sirt1 (*Ps* < 0.01) (Figures 6A–D).

## 4. Discussion

Studies have shown that rodents that experience early life stress commonly exhibit behavioral abnormalities in adulthood, including anxiety- and depression-like behaviors (Godoy et al., 2018). In the present study, we found that maternal separation led to anxiety- and depression-like behaviors in offspring, the upregulation of the hippocampal levels of pro-inflammatory cytokines (IL-1β, IL-6, and TNF-α), and the inhibition of the Sirt1/NF-κB signaling pathway. Importantly, our results showed that resveratrol could reverse these maternal separation-induced effects through the upregulation of the Sirt1/NF-κB pathway.

### 4.1. Resveratrol alleviated anxiety- and depression-like behaviors induced by maternal separation

In modern society, mother-child relationships can be adversely affected by a variety of social and environmental factors. Disruption of the normal mother-offspring relationship during a critical period of brain development can have deleterious effects on the behavior and physiological function of the offspring (Bergman, 2019). Mother-offspring separation, a widely employed rodent model of early life stress, could dysfunctions the hypothalamic-pituitary-adrenal (HPA) axis, serotonergic, and dopaminergic activities in offspring by depriving them of an appropriate early life environment, which lead to persistent changes in the neurobiology and emotional-related behaviors in adulthood (Nishi et al., 2014; Zhang X. et al., 2020; Tsotsokou et al., 2021). Studies have shown that prolonged maternal separation (3 h per day) accentuates depressive-like behaviors in adult male C57/BL6J mice in the forced swimming test (Zhou et al., 2020) and anxiety-like behaviors in adolescent male Sprague-Dawley rats as determined in the open field and Y-maze tasks (Zhang X. et al., 2020). Consistent with these observations, our results showed that adult male mice exposed to maternal separation in early life exhibited anxiety-like behaviors, as evidenced by a decrease in the time spent in and the number of entries into the central region in the open field test and a decrease in the time spent in and the number of entries into the open arms in the elevated plus maze test relative to animals in the CON group. These mice also exhibited depression-like behaviors, as indicated by an increase in immobility time in the forced swimming and tail suspension tests. However, the results of studies on the effects of maternal separation on anxiety- and depression-like behaviors in adulthood have been contradictory, with some reporting no alterations in behaviors, or even a reduction in anxiety- and depression-like behaviors in different protocols. For example, brief maternal separation (15 min per day) was reported to reduce both anxiety- and depression-like behaviors in female C57BL/6J mice with imiquimod-induced psoriasis as evaluated using the sucrose preference and open field tasks (Zhou et al., 2022). Additionally, adult male BALB/c mice that underwent maternal separation for 4 h per day were categorized as exhibiting anxiolytic behavior in the elevated plus maze and marble-burying tests (Jarrar et al., 2022). These heterogeneous results may stem from differences in the duration and number of days of the maternal separation or differences in the mouse strains used. Notably, the maternal separation paradigm of 4 h daily for 20 days used in our experiments is based on our pre-experiments and the study by Huang et al., in which not only did the male offspring mice exhibit increased anxiety-like behavior but also increased depression-like behavior (Huang et al., 2021). In the future, more researches are needed to standardize the maternal separation paradigm that meets the behavioral phenotypic changes we expect and to clarify their potential mechanisms.

Numerous studies have focused the pathophysiological processes of anxiety and depression disorders on impairments in the monoamine transmission system (Liu et al., 2015; Grinchii and Dremencov, 2020; Roman and Irwin, 2020; Falk et al., 2023). Selective serotonin reuptake inhibitors (SSRIs), selective serotonin and noradrenalin reuptake inhibitors (SNRIs) have been widely used to treat patients with anxiety or depression disorders, via enhancing monoamine function by specifically inhibiting the reuptake of these neurotransmitters in the brain (Kenda et al., 2022). However, these anxiolytics and antidepressants may frequently produce side effects, including nausea, vomiting, insomnia, headaches, and sexual dysfunction (Stahl, 1998; Yeung et al., 2018). This makes an urgent need to acquire new remedy that easily available and mild side effects, and the diversity of neural targets makes plant-derived remedy, such as resveratrol, for a promising candidate to treat these psychiatric disorders. Preclinical studies have shown that resveratrol plays an antidepressant and anxiolytic role in rodent models of estrogen deficiency-induced anxiety and depression and chronic normobaric hypoxia -induced anxiety (Fan et al., 2018; Liu et al., 2019). Our findings suggested that resveratrol improved anxiety-like behaviors caused by maternal separation, as evidenced by the significant increase in the time spent in and the number of entries into the central region in the open field test, and the increase in the time spent in and the number of entries into the open arms in the elevated plus maze. Furthermore, resveratrol reduced the immobility time of maternally separated mice in the forced swimming and tail suspension tests, implying that resveratrol ameliorated depression-like behaviors caused by maternal separation.

### 4.2. Effect of resveratrol on inflammation from maternal separation was mediated via Sirt1/NF-κB signaling pathway

Accumulating evidence suggests that inflammation can lead to neuronal apoptosis, synaptic remodeling, and alterations in the neuroendocrine system, which, in turn, can result in anxiety and depression (Morris et al., 2015; Ransohoff, 2016; Gopinath et al., 2022). The inflammatory hypothesis of anxiety and depression is supported by both clinical and preclinical studies. Patients with depression disorder exhibit a chronic inflammatory state which is characterized by elevated serum levels of pro-inflammatory cytokines, including TNF-α, IL-1β, IL-2, IL-6, IL-12, and decreased levels of anti-inflammatory cytokines, such as IL-10, compared to controls (Petralia et al., 2020). The relationship between mood disorder and inflammation has also been explored in several mouse models. For example, preclinical studies showed that Knockout (KO) mice with TNF-α receptor 1 or 2 (TNFR1-2 KO) showed increased depression-like behaviors compared to wild-type mice (Ma et al., 2016) while lateral ventricle injection of TNF-α in mice can induced mood disorders (Haji et al., 2012). Studies show that stress-induced neuroinflammation could damage hippocampal neurogenesis and contribute to emotional and cognitive-behavioral deficits (McKim et al., 2016). Therefore, it is interesting to focus on the role that inflammation plays in stress-induced anxiety and depression disorders. Maternal separation has been reported to increase the expression of pro-inflammatory cytokines accompanied by anxiety and depressive symptoms in Wistar rats offspring (Wang et al., 2020). Consistent with these reports, we found that anxiety- and depression-like behaviors in maternally separated male C57BL/6J offspring were accompanied by an increase in the expression of the pro-inflammatory cytokines IL-1β, IL-6, and TNF-α in the hippocampus.

Sirt1 is a nicotinamide adenine dinucleotide (NAD^+^)-dependent protein deacetylase with reported anti-inflammatory, anti-oxidative stress, anti-aging, and neuroprotective properties (Sakr et al., 2015). The level of Sirt1 expression is closely associated with anxiety and depression (Abe-Higuchi et al., 2016; Yu et al., 2018). Hippocampal Sirt1 expression levels are reduced in sleep deprivation-induced pathological models, resulting in anxiety- and depression-like behavioral phenotypes in Wistar male rats (Kang et al., 2021). Substantial evidence supports that the activation of Sirt1 can improve anxiogenic and depressive symptoms by inhibiting multiple pro-inflammatory signaling pathways, including the NF-κB pathway (Bian et al., 2022).

The NF-κB protein complex is a key regulator of the expression of inflammation-related genes, and its activation is closely associated with several neuropsychiatric disorders, including depression (Yang et al., 2019). In addition, it has been shown that activation of NF-κB in the hippocampus can lead to microglia activation and inflammatory responses (Zhang W. Y. et al., 2020). The post-translational modification of NF-κB p65, such as its acetylation, plays a vital role in regulating NF-κB activation. For instance, the acetylation of NF-κB p65 at lysine residue 310 is necessary for the transactivation of the NF-κB (Kauppinen et al., 2013). Therefore, Sirt1 could reduce the abundance of acetylated NF-κB p65 by deacetylation, consequently, inhibiting the transcriptional effects of NF-κB on downstream factors such as IL-1β, IL-6, and TNF-α (Yeung et al., 2004). It has been shown that via activating the Sirt1/NF-κB pathway resveratrol could attenuate inflammatory responses and anxiety-like behaviors induced by chronic normobaric hypoxia in male C57BL/6 mice (Fan et al., 2018). Similarly, the alleviating effects of neuroinflammation and depression were obtained through activating the Sirt1/NF-κB signaling pathway by Polydatin in lipopolysaccharide-treated mice (Bian et al., 2022). In the present study, we found that maternal separation downregulated the levels of Sirt1 and elevated those of NF-κB p65 and acetylated NF-κB p65, effects that could be reversed by the administration of resveratrol. These findings suggested that resveratrol can ameliorate maternal separation-induced inflammation by activating the Sirt1/NF-κB signaling pathway.

The present study had several limitations. First, studies have shown that the effects of maternal separation on offspring emotions can differ between sexes (Francis-Oliveira et al., 2021). However, we only assessed the effect of resveratrol on maternal separation-induced anxiety- and depression-like behaviors in the male offspring, as fluctuating hormone levels in female mice may affect the results of behavioral experiments (Wei et al., 2018; Kim et al., 2022). Secondly, we only examined the changes in the expression of inflammatory factors and the Sirt1/NF-κB signaling pathway in hippocampal regions and did not evaluate the changes in these indicators in other anxiety- and depression-related brain regions, such as the amygdala. Thirdly, we did not use Sirt1 blockers to inhibit the Sirt1/NF-κB signaling pathway, and thus did not investigate potential behavioral changes in maternally separated offspring or the effects of resveratrol administration under these conditions.

## 5. Conclusion

The results of this study suggested that maternal separation can lead to inflammation response and anxiety- and depression-like behavior in male offspring, effects that are mediated by the downregulation of the Sirt1/NF-κB signaling pathway. Resveratrol ameliorated inflammation and anxiety- and depression-like behaviors induced by maternal separation by activating Sirt1 and thereby inhibiting the NF-κB signaling pathway. Our findings further highlight the potential of Sirt1 as a novel target for the treatment of anxiety and depression.

## Data availability statement

The raw data supporting the conclusions of this article will be made available by the authors, without undue reservation.

## Ethics statement

This animal study was reviewed and approved by the Association of Laboratory Animal Sciences and the Center for Laboratory Animal Sciences at Anhui Medical University (approval number: LLSC20190710).

## Author contributions

R-MW and Y-MZ conceived and designed the study and drafted the manuscript. Y-ZF and K-XZ carried out the ELISA, western blotting, and RT-PCR. J-YZ, JC, and B-LL performed the behavioral experiments and participated in the study design and statistical analysis. G-HC and X-YL managed the study, revised the manuscript, and take responsibility for the integrity of the data and the accuracy of the data analysis. All authors read and approved the final version of the manuscript.

## Footnotes

1
https://www.frontiersin.org/guidelines/policies-and-publication-ethics
